# Evaluating Sepsis Management and Patient Outcomes: A Comprehensive Retrospective Study of Clinical and Treatment Data

**DOI:** 10.3390/jcm14103555

**Published:** 2025-05-19

**Authors:** Sahbanathul Missiriya Jalal, Suhail Hassan Jalal, Abeer Abbas Alabdullatif, Kamilah Essa Alasmakh, Zahraa Hussain Alnasser, Wadiah Yousef Alhamdan

**Affiliations:** 1 Department of Nursing, College of Applied Medical Sciences, King Faisal University, Al-Ahsa 31982, Saudi Arabia; 2 Department of Pharmacy, Jaya College of Pharmacy, The Tamil Nadu Dr. MGR Medical University, Chennai 602024, India

**Keywords:** sepsis, sepsis management, patient outcomes, antibiotic therapy, retrospective study, SOFA score

## Abstract

**Background/Objectives:** Sepsis, as a major cause of mortality worldwide, requires timely diagnosis and prompt treatment to improve patient outcomes. In this study, we evaluated sepsis management strategies and their impact on clinical outcomes in hospitalized patients. **Methods:** A retrospective study was conducted by analyzing clinical and treatment data from the electronic records of sepsis patients who had been admitted to tertiary care hospitals in eastern Saudi Arabia. Using systematic sampling, the details of eligible patients were obtained. Data were collected on patient demographics, vital signs, Sequential Organ Failure Assessment (SOFA) and laboratory parameters, treatment (antibiotic therapy, vasopressor use, or fluid resuscitation), and outcomes (survival in hospital). Statistical analyses were performed to assess the association between clinical and treatment strategies and patient outcomes. **Results:** A total of 234 sepsis cases were analyzed, of which 70.9% were survivors and 29.1% were non-survivors. Patients aged 60 years and above were the most affected. Statistically significant differences were observed across all of the measured vital sign variables and outcomes (*p* < 0.0001). Based on SOFA scores, 56.41% of patients were assessed as having a moderate risk. Through our comparison of clinical and laboratory parameters between survivors and non-survivors, significant differences were found in all of the measured variables (*p* < 0.0001). The odds of survival were significantly higher in those who received early administration of broad-spectrum antibiotics (OR = 4.9449, *p* = 0.0001), vasopressor therapy (OR = 1.9408, *p* = 0.0262), and fluid resuscitation OR = 11.035, *p* = 0.0001). **Conclusions:** The results of this study highlight the importance of early sepsis recognition, prompt antibiotic therapy, and standardized protocol adherence in improving patient outcomes and reducing mortality and morbidity.

## 1. Introduction

Sepsis, a life-threatening form of organ dysfunction resulting from the body’s dysregulated immune response to infection, leads to systemic inflammation, endothelial dysfunction, and multi-organ failure. This condition remains a critical challenge in healthcare settings worldwide [1]. The most common sources of infection leading to sepsis include pneumonia (40–50%), intra-abdominal infections (20–25%), and urinary tract infections (10–15%) [2]. The immune response to infection triggers the release of pro-inflammatory cytokines, such as tumor necrosis factor-alpha (TNF-α), interleukin-1β (IL-1β), and interleukin-6 (IL-6), leading to widespread tissue damage, capillary leakage, and hypoperfusion [3].

Despite advancements in medical science, sepsis continues to be associated with high morbidity and mortality rates, imposing significant burdens on healthcare systems [4]. Understanding the complexities of sepsis management and its impact on patient outcomes is paramount for developing effective therapeutic strategies and improving survival rates [2]. On an annual basis, sepsis affects millions globally, with a substantial number of cases leading to death or long-term disability.

In the United States alone, sepsis accounts for more than 50% of hospital deaths, underscoring the severity of this condition and the urgency of timely intervention [5]. Sepsis accounts for at least 1.7 million adult hospitalizations annually, leading to approximately 350,000 deaths each year [6]. The economic implications are equally alarming, with sepsis management costs ranking among the highest for hospital admissions across various illnesses. These statistics highlight the pressing need for enhanced sepsis recognition and management protocols to mitigate its devastating impact [7].

While a considerable body of sepsis research focuses on global data, in the present study, we highlight the burden and mortality of sepsis in eastern Saudi Arabia, a region with limited published epidemiological data. Approximately 128,000 cases of sepsis are reported annually, resulting in roughly 64,000 sepsis-related deaths [8]. The authors of a study conducted at Buraidah Central Hospital in Qassim reported that 16% of intensive care unit (ICU) admissions were a direct result of severe sepsis, with a mortality rate of 40.3% among these patients. These statistics underscore the critical need for enhanced sepsis awareness, early detection (within the first 1–3 h), and effective management strategies within the Saudi healthcare system to reduce the incidence and mortality associated with this life-threatening condition [9].

Timely identification of sepsis is crucial for initiating appropriate therapeutic measures. However, the clinical presentation of sepsis is often heterogeneous, complicating early diagnosis [10]. The Sequential Organ Failure Assessment (SOFA) score is a widely implemented clinical assessment tool used to evaluate organ dysfunction in sepsis, which includes assessing respiratory rate, altered mental status, and systolic blood pressure. The SOFA score is a more reliable indicator for identifying and diagnosing septic patients [11,12]. The implementation of structured sepsis management protocols has been a focal point in improving patient outcomes [13]. Early intervention (within 3 h) is a cornerstone in treating sepsis, with prompt administration of antibiotics and supportive care significantly influencing patient outcomes. Sepsis management is complex, with there being an urgent need for continuous evaluation of clinical protocols to identify the measures necessary to improve survival [14].

Despite advancements in sepsis care, significant challenges remain. Antibiotic resistance, particularly in multidrug-resistant (MDR) organisms, complicates treatment and increases mortality risk. The authors of several studies have reported a rise in carbapenem-resistant Klebsiella pneumoniae and Acinetobacter baumannii in intensive care unit (ICU) patients with sepsis, necessitating new antimicrobial programs to guide appropriate antibiotic use [15]. Another major challenge is the variability in sepsis recognition and adherence to treatment guidelines. Considering these challenges, we aimed to evaluate sepsis management strategies in the present study and their impact on patient clinical outcomes.

## 2. Materials and Methods

### 2.1. Study Design and Setting

In this study, we adopted a retrospective cohort design to evaluate sepsis management and patient outcomes based on clinical and treatment data. This study involves the analysis of the electronic health records (EHRs) of patients admitted to various healthcare facilities with sepsis from January to December 2024. The study was conducted in selected tertiary care hospitals in eastern Saudi Arabia.

### 2.2. Sample Size and Sampling

**Figure 1:**
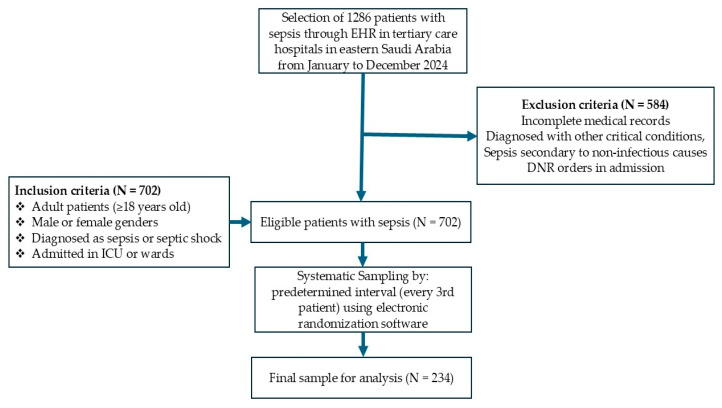
Flowchart of patient inclusion in this study.

In this retrospective study, the sample size was determined using the formula (*n* = (Z^2^ × p × (1 − p))/d^2^) to be approximately 207, with a margin of error of 5%, a confidence level of 95%, and an estimated prevalence of sepsis in the target population of around 16%, based on the results of a previous study [9]. To enhance the study’s statistical power and account for missing data, a total of 234 patients were selected, as illustrated in Figure 1. A systematic sampling technique was applied to select eligible patients from the selected hospital records. The procedure involved the following stages: extracting a list of all sepsis patients from the hospital database meeting the inclusion criteria, assigning a unique identifier to each patient, using a systematic selection method to choose 234 patients from the total eligible population, and ensuring an unbiased selection process by using predetermined intervals.

### 2.3. Inclusion and Exclusion Criteria

The collected data included those of adult patients (≥18 years old), either male or female, diagnosed with sepsis or septic shock according to the sepsis-3 criteria, patients admitted to the intensive care unit (ICU) or general wards with documented sepsis management protocols, and those with availability of complete clinical and treatment records. Patients with incomplete medical records, those diagnosed with other critical conditions or sepsis secondary to non-infectious causes, and those with do not resuscitate (DNR) orders at the time of admission were excluded from the study.

### 2.4. Data Collection and Tool

Data were extracted by using the patients’ EHRs, with the utilization of a structured data collection tool from January to December 2024, to identify patients admitted to the hospital and diagnosed with sepsis. To maintain privacy and confidentiality, the collected data were electronically encrypted and stored securely, with access restricted to the principal investigator through a password. The evaluation consisted of five variables, namely, demographics, vital signs, SOFA and laboratory parameters, treatment, and outcomes.

#### 2.4.1. Demographic Variables

The first variable details demographic data, including age, gender, cause of sepsis, such as respiratory infections, urinary tract infections, abdominal infections, bloodstream infections, skin and soft tissue infections, and other infections, length of hospital stay, and 28-day mortality.

#### 2.4.2. Vital Signs

The second variable concerns patients’ vital signs, including temperature, heart rate, respiratory rate, systolic blood pressure (SBP), diastolic blood pressure (DBP), and blood oxygen saturation (SpO_2_) level. Respiratory rates were collected after mechanical ventilation was initiated in patients requiring this treatment; however, not all patients were ventilated, leading to some heterogeneity in the data. SpO_2_ values were recorded under varying oxygen delivery methods and concentrations, depending on the patient’s clinical status. The acute physiology and chronic health evaluation II (APACHE II) score was calculated based on the worst physiological values recorded within the first 24 h of ICU admission, in accordance with standard scoring guidelines. Relevant parameters, including systolic and diastolic blood pressure, SpO_2_, and PaO_2_, were extracted retrospectively from electronic medical records and ICU monitoring charts. For each patient, the most clinically significant (i.e., the most variable) value observed during the initial 24 h period was used. This approach was uniformly applied across all physiological variables to ensure consistency and accuracy. To minimize bias, those involved in the data collection process were blinded to outcome statuses, and APACHE II scoring was independently performed by two trained clinicians. These measures were employed to avoid selection bias and maintain the objectivity and reliability of the scoring process.

#### 2.4.3. SOFA Scores

The third variable comprises the SOFA score, including the Glasgow coma scale (GCS), arterial pressure or administration of vasopressors required, partial pressure of oxygen_2_ (PaO_2)_ level, and laboratory parameters, such as platelet, bilirubin, and creatinine levels. Depending on the clinical state of the patient, PaO_2_ values were collected using various oxygen delivery techniques and under different concentrations. Each organ system was assigned a score from 0 (normal function) to 4 (severe dysfunction/failure) based on specific clinical and laboratory parameters. The total SOFA score ranges from 0 to 24. A total score between 0 and 6 indicates a low risk of organ dysfunction or failure, a score between 7 and 9 indicates a moderate risk of organ dysfunction, a score between 10 and 12 indicates a high risk of organ failure and poor prognosis, and a score above 12 indicates severe multi-organ failure with high mortality risk [16].

#### 2.4.4. Treatment Strategies

The fourth variable relates to treatment strategies, which included the administration of antibiotics, initiation and duration, fluid resuscitation, and vasopressor use. In this study, “early” means administering broad-spectrum antibiotics within the first three hours after identifying sepsis or septic shock, as recommended by the Surviving Sepsis Campaign. “Broad-spectrum antibiotics” are defined as substances that are effective against a broad variety of Gram-positive and Gram-negative organisms. These organisms generally include carbapenems, third- or fourth-generation cephalosporins, or combined therapy using substances like piperacillin–tazobactam and vancomycin. Fluid resuscitation was considered to have been implemented if there was documented administration of an initial bolus of crystalloid fluids within the first 3 h after sepsis recognition, again following sepsis management guidelines. The patients were at various stages of sepsis and septic shock management at the time of measurement, and vasopressor doses were not standardized across cases. Vasopressors were administered to patients who remained hypotensive with low mean arterial pressure (MAP) despite initial adequate fluid resuscitation, following standard sepsis protocols. For cases in which vasopressors were not used despite the presence of hypotension, this choice was often based on physician judgment, patient comorbidities, goals established during care discussions, or resource limitations.

#### 2.4.5. Outcomes of Sepsis

The fifth variable refers to the outcomes of sepsis patients, including the survival of patients during hospital stays.

### 2.5. Ethical Considerations

Ethical clearance was obtained from the Research Ethics Committee of the Deanship of Scientific Research, King Faisal University, Saudi Arabia (KFU-REC-2024-NOV-ETHICS2896) dated 27 November 2024, and the study adhered to the guidelines of the Declaration of Helsinki. Ethical approval was also secured from the institutional review board of the hospitals. After obtaining permission from the medical record authority, EHRs were accessed, and the collected data were anonymized, kept securely, and stored confidentially.

### 2.6. Statistical Analysis

Statistical analysis was performed using SPSS software (version 21.0; IBM, Armonk, NY, USA) to evaluate the data collected in the study. For the descriptive analysis, continuous variables were expressed as mean ± standard deviation (SD), while categorical variables were presented as frequencies and percentages. For the comparative analysis, student’s *t*-test was used for continuous variables, and the chi-square test was applied for categorical variables. For the survival analysis, the odds ratio test was used to assess survival differences between treatment groups. A *p*-value of 0.05 or less was considered statistically significant.

## 3. Results

### 3.1. Demographic Variables of Patients with Sepsis

A total of 234 septic patients were included in the analysis (Table 1), of which 166 (70.9%) were survivors and the remaining 68 (29.1%) were non-survivors. Age distribution showed that 179 (76.5%) patients aged 60 years and above were the most affected. In contrast, 13 (5.6%) patients in younger age groups (19–39 years) had fewer sepsis cases, although the association between age and progression of sepsis was not statistically significant (*p* = 0.2285). The mean age was 65.55 (SD ± 12.9) years. In terms of gender, among 150 (64.1%) males, most of them, 107 (45.7%), were survivors, and the remaining 43 (18.4%) were non-survivors. Among females, 59 (25.2%) accounted for survivors, and the remaining 25 (10.7%) were non-survivors. However, gender did not show a significant association with sepsis (*p* = 0.8595).

Respiratory infections were the leading cause of sepsis, where 66 (28.2%) cases were survivors and 23 (9.8%) were non-survivors of sepsis. Urinary tract infections were the second most common cause, of which 39 (16.7%) were survivors and 18 (7.7%) were non-survivors, followed by abdominal infections, of which 26 (11.1%) patients with sepsis were survivors and 12 (5.1%) were non-survivors. Bloodstream infections, skin and soft tissue infections, and other infections were relatively less frequent. However, there was no statistically significant association between the cause of sepsis and progression to septic shock (*p* = 0.9408). A significant difference was observed in hospital stay duration among patients with sepsis. Approximately 96 (41%) patients with hospital stays exceeding 28 days were more likely to be survivors and 22 (9.4%) were non-survivors compared to those hospitalized for less than 28 days. This association was statistically significant (*p* = 0.0004), suggesting prolonged hospital stay as a potential risk factor for sepsis mortality.

### 3.2. Physiological Parameters of Patients with Sepsis

The comparison of physiological parameters between the survivor and non-survivor groups revealed that there were statistically significant differences across all of the measured vital sign variables (Table 2). The mean body temperature in the survivor group was 37.53 ± 0.33 °C, while in the non-survivor group it was 38.06 ± 0.31 °C. The difference was highly significant (*p* < 0.0001). In the non-survivor group, a higher mean heart rate (107.78 ± 17.76 bpm) was exhibited compared to the survivor group (93.86 ± 19.57 bpm), which was also highly significant (*p* < 0.0001). The mean respiratory rate was slightly lower in the non-survivor group (20.6 ± 5.11 breaths per minute) compared to the survivor group (22.92 ± 6.96 breaths per minute), with a statistically significant difference (*p* = 0.0136). The survivor group had a significantly higher mean systolic blood pressure (128.55 ± 19.93 mmHg) compared to the non-survivor group (120.32 ± 25.48 mmHg), which showed a statistically significant difference (*p* = 0.0089). Similarly, the mean diastolic blood pressure was higher in the survivor group (79.05 ± 12 mmHg) than in the non-survivor group (74.56 ± 10.37 mmHg), indicating a significant difference (*p* = 0.0074). The survivor group had a slightly higher mean oxygen saturation (97.11 ± 1.82%) compared to the non-survivor group (96.29 ± 1.85%) (*p* = 0.0021), indicating a statistically significant but clinically modest difference.

### 3.3. SOFA Score of Patients with Sepsis

**Figure 2:**
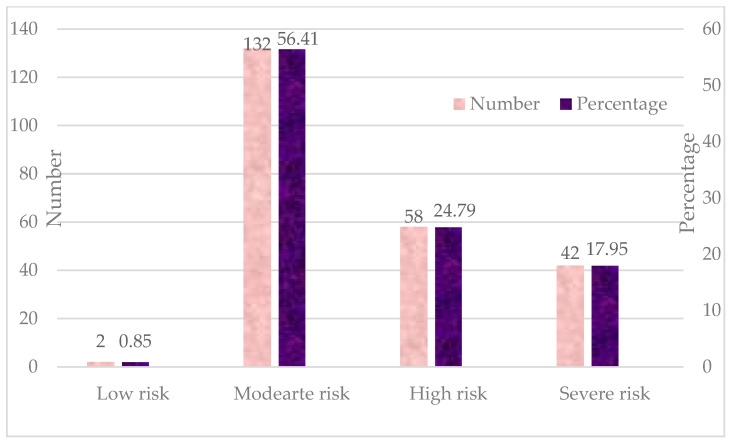
Frequency distribution of SOFA score of patients with sepsis (*n* = 234).

As per the SOFA score, the risk classifications of sepsis patients are depicted in Figure 2. Only two patients (0.85%) fell into the low-risk category, indicating minimal organ dysfunction and a low likelihood of adverse outcomes. Most of the patients, 132 (56.41%), were classified as having a moderate risk, suggesting some degree of organ dysfunction but a relatively favorable prognosis. Around 58 patients (24.79%) were classified as high risk, indicating significant organ dysfunction and an increased probability of severe complications or mortality. Approximately 42 patients (17.95%) fell into the severe risk category, reflecting critical organ failure and a high mortality risk.

The comparison of clinical and laboratory parameters between survivors and non-survivors revealed significant differences in all measured variables (Table 3). Survivors had a significantly higher mean GCS score (13.02 ± 1.17) compared to non-survivors (8.32 ± 1.55) (t = 25.285, *p* = 0.0001), indicating worse neurological status among non-survivors. The mean arterial pressure was significantly lower in non-survivors (60.71 ± 7.83 mmHg) compared to survivors (64.75 ± 4.33 mmHg) (t = 5.0342, *p* = 0.0001), suggesting hemodynamic instability in the non-survivor group. Survivors had a significantly higher PaO_2_ (289.35 ± 11.48 mmHg) compared to non-survivors (241.97 ± 56.66 mmHg) (t = 10.3, *p* = 0.0001), indicating poorer oxygenation in the non-survivor group. The mean platelet count was significantly lower in non-survivors (100.53 ± 23.7 ×10^3^/μL) than in survivors (125.53 ± 14.14 ×10^3^/μL) (t = 9.9533, *p* = 0.0001), suggesting greater coagulopathy in the non-survivor group. Non-survivors had significantly higher bilirubin levels (4.74 ± 1.67 mg/dL) than survivors (2.68 ± 0.89 mg/dL) (t = 12.2259, *p* = 0.0001), indicating more severe liver dysfunction in the non-survivor group. Non-survivors had significantly higher serum creatinine levels (3.11 ± 0.71 mg/dL) compared to survivors (2.32 ± 0.47 mg/dL) (t = 9.8888, *p* = 0.0001), reflecting worse renal function in the non-survivor group. The mean SOFA score was significantly higher in non-survivors (12.84 ± 1.49) compared to survivors (8.59 ± 0.95) (t = 26.0598, *p* = 0.0001), confirming greater organ dysfunction and a worse prognosis in non-survivors.

### 3.4. Treatment Strategies for Patients with Sepsis

The analysis of different treatment strategies between survivors (*n* = 166) and non-survivors (*n* = 68) revealed statistically significant associations with survival outcomes (Table 4). Among survivors, 127 (54.3%) received early broad-spectrum antibiotics compared to only 27 (11.5%) non-survivors. The odds of survival were significantly higher in those who received early broad-spectrum antibiotics (OR = 4.9449, 95% CI: 2.7032–9.0457, Z = 5.187, *p* = 0.0001). Regarding administering antibiotics based on the culture and sensitivity test results, 134 (57.3%) survivors received culture-based antibiotic therapy, compared to 25 (10.7%) non-survivors. The odds of survival were significantly higher in patients who received antibiotics based on culture results (OR = 7.2025, 95% CI: 3.8518–13.468, Z = 6.183, *p* = 0.0001). A higher percentage of survivors (118, 50.4%) received vasopressors compared to 38 (16.2%) non-survivors. Vasopressor support was associated with increased survival (OR = 1.9408, 95% CI: 1.0817–3.4822, Z = 2.223, *p* = 0.0262), although the effect was weaker compared to antibiotics or fluid resuscitation. Fluid resuscitation was received by 141 (60.3%) survivors compared to only 23 (9.8%) non-survivors. The odds of survival were significantly higher in patients who received fluid resuscitation (OR = 11.035, 95% CI: 5.7133–21.313, Z = 7.149, *p* = 0.0001), suggesting a strong protective effect.

## 4. Discussion

Sepsis remains a critical condition characterized by high morbidity and mortality, particularly among older adults and those with comorbid infections. In this study, we analyzed the demographic variables associated with sepsis outcomes, including age, gender, infection source, and hospital stay duration. The results indicated that older adults (≥60 years) were the most affected by sepsis, accounting for 76.5% of cases. This finding aligns with those of previous studies, suggesting that aging is a significant risk factor for sepsis due to the presence of immunosenescence, comorbidities, and altered physiological responses to infection [17,18]. Despite the high prevalence of sepsis in elderly patients, however, we did not find a statistically significant association between age and sepsis progression. The authors of another study in this field suggested that while age increases susceptibility to sepsis, other factors, such as underlying health conditions and infection severity, may play a greater role in determining outcomes [19]. Gender distribution showed that males (64.1%) were more frequently affected by sepsis than females (35.9%), which is consistent with the results of previous studies indicating that male patients tend to have a higher incidence of sepsis due to immune response differences and comorbidities [20,21].

In the present study, respiratory infections were the most common cause of sepsis, followed by urinary tract infections and abdominal infections. This distribution aligns with global epidemiological trends showing that pneumonia and urinary tract infections are the leading causes of sepsis in hospitalized patients [22,23]. A significant difference was observed in hospital stay duration between survivors and non-survivors. Patients with hospital stays exceeding 28 days were more likely to survive; in comparison, shorter hospital stays were associated with higher mortality (*p* = 0.0004). This finding is consistent with those of studies indicating that prolonged hospital stays may be a marker of aggressive treatment and better supportive care, enabling critically ill patients to recover over time [24]. However, prolonged hospitalization can also lead to secondary infections, antibiotic resistance, and complications, increasing the risk of adverse outcomes [25].

In this study, the comparison of physiological parameters between survivors and non-survivors revealed significant differences across all measured vital signs, including temperature, heart rate, respiratory rate, BP, and oxygen saturation, which highlights their potential prognostic value in sepsis outcomes. The results of the present study demonstrate a significant difference in mean body temperature between the survivor and non-survivor groups, with non-survivors showing higher mean body temperatures (38.06 ± 0.31 °C) than survivors (37.53 ± 0.33 °C, *p* < 0.0001). This finding suggests that elevated body temperature may be associated with increased mortality risk. Early detection and management of hyperthermia may improve outcomes. The authors of previous studies have concluded that while fever is a regulated response with variable effects, in sepsis, mortality shows a tendency to vary accordingly. It should be noted, however, that both extreme hypothermia and hyperthermia are harmful conditions [26]. Conversely, an inadequate febrile response or hypothermia has been linked to worse prognosis and higher mortality rates [27]. A significantly higher mean heart rate was reported in non-survivors (107.78 ± 17.76 bpm) compared to survivors (93.86 ± 19.57 bpm, *p* < 0.0001). This finding suggests that tachycardia may be associated with increased mortality risk. An elevated heart rate often reflects an underlying response to systemic stress, such as infection, hypoxia, or shock, which are common in critically ill patients [28].

Although patients in both groups exhibited tachypnea, patients in the survivor group were found to have a slightly higher respiratory rate in this study. Increased respiratory effort in survivors may reflect a more effective compensatory response to metabolic acidosis and hypoxia. Non-survivors, in contrast, may have developed respiratory failure, which can lead to inadequate ventilation, hypercapnia, and, ultimately, worse outcomes [29]. Survivors demonstrated significantly higher mean systolic (128.55 ± 19.93 mmHg vs. 120.32 ± 25.48 mmHg, *p* = 0.0089) and diastolic blood pressure (79.05 ± 12 mmHg vs. 74.56 ± 10.37 mmHg, *p* = 0.0074) compared to non-survivors. Sepsis-induced hypotension is a well-established risk factor for mortality, often necessitating aggressive fluid resuscitation and vasopressor therapy [30]. The higher blood pressure noted in survivors suggests better vascular tone, effective fluid management, and an adequate response to treatment; in comparison, non-survivors likely experienced refractory hypotension leading to organ failure. A small but statistically significant difference in oxygen saturation was observed between survivors and non-survivors. Even slight impairments in oxygenation could contribute to worse sepsis outcomes. Hypoxia in sepsis is often a marker of respiratory failure, metabolic dysfunction, or tissue hypoperfusion, all of which contribute to increased mortality [31].

The SOFA score is a well-established tool for assessing organ dysfunction in sepsis, and the results of the present study confirm its strong prognostic value in predicting mortality risk among septic patients. The classification of patients into different SOFA risk categories demonstrated a progressive increase in mortality risk with worsening organ dysfunction. Most of the septic patients in our study fell into the moderate (56.41%) and high risk (24.79%) categories. Patients in the severe risk category (17.95%) were found to have the highest mortality rates, aligning with the results presented in the existing literature demonstrating that a SOFA score ≥10 is loosely associated with a higher likelihood of death in sepsis [16]. This classification system can be valuable for early triaging and guiding treatment intensity in critically ill patients. In this study, a significantly lower GCS score was noted in non-survivors, highlighting the impact of sepsis on neurological function. Septic encephalopathy is a common consequence of systemic inflammation, cerebral hypoxia, and metabolic disturbances, contributing to worse outcomes [32].

Septic shock, characterized by profound hypotension despite adequate fluid resuscitation, is a well-known driver of multi-organ failure and mortality [33]. This finding reinforces the importance of early vasopressor therapy (within 3 h) and fluid resuscitation to maintain adequate perfusion. Non-survivors were found to have significantly lower PaO_2_ levels, reflecting more severe respiratory impairment. This finding aligns with those of previous studies showing that hypoxemia and acute respiratory distress syndrome (ARDS) are associated with poor sepsis outcomes [34]. In this study, the lower platelet counts in non-survivors indicated more severe coagulopathy, which is a hallmark of sepsis-associated disseminated intravascular coagulation (DIC) [35]. The significantly higher bilirubin levels in non-survivors indicated more profound hepatic dysfunction, likely due to sepsis-induced cholestasis or ischemic liver injury [36]. Similarly, non-survivors were found to have significantly higher serum creatinine levels, reflecting more severe acute kidney injury (AKI), a well-known predictor of poor outcomes in sepsis [37].

The results of our analysis highlight the critical role of timely and appropriate treatment strategies in determining sepsis survival outcomes, particularly the administration of early broad-spectrum antibiotics, culture-based antibiotic selection, vasopressor support, and fluid resuscitation. The significantly higher survival rates among patients who received early broad-spectrum antibiotics (54.3% vs. 11.5%) emphasize the life-saving impact of prompt antimicrobial therapy in sepsis. The odds of survival were nearly five times higher (OR = 4.9449, *p* = 0.0001) in patients who received early administration of antibiotics, reinforcing the well-established principle that delays in antibiotic administration are associated with increased mortality in sepsis and septic shock [38]. Early empirical therapy targets the most likely pathogens before culture results are available, reducing bacterial burden and preventing progression to multi-organ failure.

Patients who received antibiotics based on culture and sensitivity testing were found to have a significantly higher survival rate. In these patients, the odds of survival were even greater (OR = 7.2025, *p* = 0.0001), suggesting that de-escalation to targeted therapy based on microbiological evidence is beneficial. These findings align with those of previous studies showing that inappropriate initial antibiotic therapy is associated with higher mortality, whereas culture-guided antibiotic adjustments improve clinical outcomes and reduce antibiotic resistance [39].

Vasopressor therapy was more commonly administered in survivors than in non-survivors, with a moderate survival benefit noted (OR = 1.9408, *p* = 0.0262). This finding suggests that early vasopressor initiation in hypotensive patients is beneficial; however, its impact on survival is less pronounced than that of antibiotics or fluid resuscitation. The results of other studies have shown that delayed vasopressor administration is linked to increased mortality [40].

The strongest predictor of survival was adequate fluid resuscitation, with 60.3% of survivors receiving fluids compared to only 9.8% of non-survivors. The odds of survival were more than 11 times higher (OR = 11.035, *p* = 0.0001) in those who received aggressive fluid resuscitation. This finding reinforces the central role of goal-directed therapy in sepsis management, as fluid resuscitation aids in restoring circulatory volume, maintaining tissue perfusion, and preventing multi-organ dysfunction [41]. However, the timing, volume, and type of fluids remain areas of ongoing debate, as excessive resuscitation can lead to fluid overload and worsen outcomes.

The key strength of our study, which involved the analysis of multiple physiological parameters, laboratory biomarkers, and treatment strategies, is the fact that it offers a holistic view of sepsis management and its impact on survival. We incorporate a validated scoring system and examine the effectiveness of early antibiotic administration, culture-based therapy, vasopressor support, and fluid resuscitation, aligning with current sepsis management guidelines. Incorporation of these factors provides strong evidence supporting early intervention strategies. The findings highlight modifiable risk factors (e.g., timely antibiotic therapy and adequate fluid resuscitation) that can guide clinical decision making and improve sepsis survival rates. The novelty of the study lies in its contextual application and outcome analysis. However, certain limitations should also be noted. In this study, we relied on retrospective data collection, which may introduce selection bias and issues related to missing data. Post-discharge data, including readmission rates and functional recovery, are crucial for assessing the long-term impact of sepsis. Due to the retrospective nature of this study and constraints in the available hospital records, these outcomes were not consistently documented and could not be reliably incorporated into the analysis. The fact that the timing and adequacy of fluid resuscitation were inferred from available documentation rather than observed directly represents another limitation. Future prospective studies are essential to capture comprehensive post-discharge outcomes, including readmission rates and functional recovery, to more comprehensively understand the long-term effects of sepsis on patient health and inform clinical management strategies. Variability in biomarker testing practices was noted across the participating hospitals, particularly for procalcitonin, C-reactive protein (CRP), and interleukin-6 (IL-6), in addition to the incomplete recording of these biomarker data in patient charts. This inconsistency prevented us from reliably incorporating biomarker levels into our analysis. The inclusion of such biomarkers will be crucial in future prospective studies, as they can provide valuable insights into the pathophysiology and progression of sepsis, enabling a more comprehensive evaluation of patient outcomes. The fact that this variability during data collection may have impacted the interpretation of the physiological parameters represents another limitation. In some cases, we found incomplete records regarding the administration of an initial bolus of crystalloid fluids.

The completion of a prospective cohort study would facilitate greater control of confounding variables and more accurate data collection. Although the treatment strategies across hospitals were compared, variations in antibiotic selection, timing of interventions, and clinical decision making may have potentially influenced the survival outcomes. The authors of future studies should include long-term outcomes, such as post-sepsis complications, quality of life, organ dysfunction recovery, and hospital readmission rates, to provide a comprehensive understanding of sepsis prognosis. Future prospective studies with standardized timing and conditions of measurement are necessary to enable a more precise analysis. Emerging technologies, particularly artificial intelligence (AI), present significant potential for transforming the early detection and management of sepsis. AI-driven tools can assist clinicians by rapidly analyzing large volumes of clinical data to identify subtle patterns indicative of sepsis earlier than can be achieved with traditional methods. Furthermore, predictive models powered by AI could aid in risk stratification, optimizing resource allocation and personalizing treatment strategies in sepsis.

## 5. Conclusions

The findings presented herein provide a comprehensive examination of patient outcomes in sepsis by comparing survivors and non-survivors based on physiological parameters, laboratory markers, SOFA scoring, and treatment strategies. The findings highlight the critical impact of early broad-spectrum antibiotic therapy, culture-guided antibiotic adjustments, vasopressor support, and fluid resuscitation on survival. The clinical and laboratory test parameters showed significant differences between survivors and non-survivors, indicating their potential role as prognostic markers in sepsis. The findings presented in this study reinforce the importance of early intervention, appropriate antimicrobial therapy, and aggressive supportive care in reducing mortality and improving patient outcomes. Overall, early recognition, timely intervention, and optimized treatment strategies remain key factors in improving survival rates among sepsis patients. The findings of this study will aid in understanding variations in practice, identifying gaps in care, and informing region-specific quality improvement initiatives.

## Figures and Tables

**Table 1:** Frequency distribution of demographic variables of patients with sepsis (*n* = 234).

Demographic Variables		Survivors (*n* = 166)	Non-Survivors (*n* = 68)	*p* Value
		N (%)	N (%)	
Age	19–39	11 (4.7)	2 (0.9)	0.2285 NS
	40–59	26 (11.1)	16 (6.8)	
	60 and above	129 (55.1)	50 (21.4)	
Gender	Male	107 (45.7)	43 (18.4)	0.8595 NS
	Female	59 (25.2)	25 (10.7)	
Cause of sepsis	Respiratory infections	66 (28.2)	23 (9.8)	0.9594 NS
	Urinary tract infections	39 (16.7)	18 (7.7)	
	Abdominal infections	26 (11.1)	12 (5.1)	
	Blood stream infections	23 (9.8)	9 (3.8)	
	Skin and soft tissue infections	7 (3.1)	4 (1.7)	
	Other infections	5 (2.1)	2 (0.9)	
Hospital stay	<28 days	70 (29.9)	46 (19.7)	0.0004 *
	>28 days	96 (41)	22 (9.4)	

* Significant (*p* < 0.05); NS—non-significant.

**Table 2:** Mean score of vital signs of patients with sepsis (*n* = 234).

Vital Signs	Mean ± SD		*p* Value
	Survivors (*n* = 166)	Non-Survivors (*n* = 68)	
Temperature (°C)	37.53 ± 0.33	38.06 ± 0.31	t = 11.2279 *p* = 0.0001 *
Heart rate (BPM)	93.86 ± 19.57	107.78 ± 17.76	t = 5.0709 *p* = 0.0001 *
Respiratory rate (breaths per minute)	22.92 ± 6.96	20.6 ± 5.11	t = 2.4863 *p* = 0.0136 *
Systolic blood pressure (mmHg)	128.55 ± 19.93	120.32 ± 25.48	t = 2.6366 *p* = 0.0089 *
Diastolic blood pressure (mmHg)	79.05 ± 12	74.56 ± 10.37	t = 2.7021 *p* = 0.0074 *
O_2_ saturation	97.11 ± 1.82	96.29 ± 1.85	t = 3.1175 *p* = 0.0021 *

* Significant (*p* < 0.05); °C—Celsius; BPM—beats per minute; mmHg—millimeter mercury.

**Table 3:** Mean score of clinical and laboratory parameters of patients with sepsis (*n* = 234).

Parameters	Mean ± SD		*p* Value
	Survivors (*n* = 166)	Non-Survivors (*n* = 68)	
GCS	13.02 ± 1.17	8.32 ± 1.55	t = 25.285 *p* = 0.0001 *
Arterial pressure (mmHg)	64.75 ± 4.33	60.71 ± 7.83	t = 5.0342 *p* = 0.0001 *
PaO_2_	289.35 ± 11.48	241.97 ± 56.66	t = 10.3 *p* = 0.0001 *
Platelet (×10^3^/μL)	125.53 ± 14.14	100.53 ± 23.7	t = 9.9533 *p* = 0.0001 *
Bilirubin (mg/dL)	2.68 ± 0.89	4.74 ± 1.67	t = 12.2259 *p* = 0.0001 *
Creatinine (mg/dL)	2.32 ± 0.47	3.11 ± 0.71	t = 9.8888 *p* = 0.0001 *
SOFA score	8.59 ± 0.95	12.84 ± 1.49	t = 26.0598 *p* = 0.0001 *

GCS—Glasgow coma scale; SOFA—Sequential Organ Failure Assessment; * significant (*p* < 0.05).

**Table 4:** Comparison of treatment of patients with outcomes of sepsis (*n* = 234).

Treatment Strategies		Survivors (*n* = 166)		Non-Survivors (*n* = 68)		*p* Value	Odds Ratio	95% CI	*p*-Value
		N	%	N	%				
Early broad-spectrum antibiotics therapy	Yes	127	54.3	27	11.5	X^2^ = 29.035 *p* = 0.0001 *	4.9449	2.7032 to 9.0457	Z = 5.187 *p* = 0.0001 *
	No	39	16.7	41	17.5				
Antibiotics based on culture test	Yes	134	57.3	25	10.7	X^2^ = 42.801 *p* = 0.0001 *	7.2025	3.8518 to 13.468	Z = 6.183 *p* = 0.0001 *
	No	32	13.7	43	18.4				
Vasopressor support	Yes	118	50.4	38	16.2	X^2^ = 5.0167 *p* = 0.0369 *	1.9408	1.0817 to 3.4822	Z = 2.223 *p* = 0.0262 *
	No	48	20.5	30	12.8				
Fluid resuscitation	Yes	141	60.3	23	9.8	X^2^ = 60.119 *p* = 0.0001 *	11.035	5.7133 to 21.313	Z = 7.149 *p* = 0.0001 *
	No	25	10.7	45	19.2				

X^2^—chi-square; Z—z-score; * significant (*p* < 0.05).

## Data Availability

Data are contained within the article.

## Abbreviations

AI	Artificial intelligence
APACHE II	Acute physiology and chronic health evaluation II
ARDS	Acute respiratory distress syndrome
BPM	Beats per minute
CI	Confidence interval
DBP	Diastolic blood pressure
DIC	Disseminated intravascular coagulation
DNR	Do not resuscitate
HER	Electronic health record
GCS	Glasgow coma scale
ICU	Intensive care unit
MAP	Mean arterial pressure
MDR	Multi-drug resistant
OR	Odds ratio
PaO_2_	Partial pressure of oxygen
SOFA	Sequential Organ Failure Assessment
SBP	Systolic blood pressure
SD	Standard deviation
SPSS	Statistical Package for Social Sciences
WHO	World Health Organization
